# Critical to Know Pcrit: A Review on Pharyngeal Critical Closing Pressure in Obstructive Sleep Apnea

**DOI:** 10.3389/fneur.2022.775709

**Published:** 2022-02-22

**Authors:** Elahe Kazemeini, Eli Van de Perck, Marijke Dieltjens, Marc Willemen, Johan Verbraecken, Sara Op de Beeck, Olivier M. Vanderveken

**Affiliations:** ^1^Faculty of Medicine and Health Sciences, University of Antwerp, Wilrijk, Belgium; ^2^Ear, Nose, Throat, Head and Neck Surgery, Antwerp University Hospital, Edegem, Belgium; ^3^Multidisciplinary Sleep Disorders Centre, Antwerp University Hospital, Edegem, Belgium; ^4^Department of Pulmonology, Antwerp University Hospital, Edegem, Belgium

**Keywords:** OSA, collapsibility, endotyping, Pcrit, upper airway

## Abstract

It is crucial to understand the underlying pathophysiology of obstructive sleep apnea (OSA). Upper airway collapsibility is an important pathophysiological factor that affects the upper airway in OSA. The aim of the current study was to review the existing body of knowledge on the pharyngeal collapsibility in OSA. After a thorough search through Medline, PubMed, Scopus, and Web of science, the relevant articles were found and used in this study. Critical closing pressure (Pcrit) is the gold standard measure for the degree of collapsibility of the pharyngeal airway. Various physiological factors and treatments affect upper airway collapsibility. Recently, it has been shown that the baseline value of Pcrit is helpful in the upfront selection of therapy options. The standard techniques to measure Pcrit are labor-intensive and time-consuming. Therefore, despite the importance of Pcrit, it is not routinely measured in clinical practice. New emerging surrogates, such as finite element (FE) modeling or the use of peak inspiratory flow measurements during a routine overnight polysomnography, may enable clinicians to have an estimate of the pharyngeal collapsibility. However, validation of these techniques is needed.

## Introduction

Within the spectrum of sleep-disordered breathing, obstructive sleep apnea (OSA) is highly prevalent (1, 2). The OSA is caused by a recurring collapse of the upper airway during sleep, resulting in complete (apnea) or partial (hypopnea) cessation of airflow (3). This leads to micro-arousals and nocturnal hypoxemia (4). The severity of OSA is expressed by the apnea-hypopnea index (AHI), defined as the number of apneas and hypopneas per hour of sleep (5).

There are five key pathophysiological factors that affect the upper airway and ventilation during sleep, with varying importance of each trait in each individual patient (6, 7): the site and pattern of the collapse in the upper airway, collapsibility of the upper airway, ventilatory control stability, pharyngeal muscle responsiveness, and arousal threshold.

Critical closing pressure (Pcrit) is the gold standard measure for the degree of collapsibility of the pharyngeal airway (8). Several methods to measure Pcrit have been published. This study focuses on the pharyngeal collapsibility and its measurement methods.

## Materials and Methods

All procedures regarding the creation of this study was in accordance with the ethical standards. A thorough search through Medline, PubMed, Scopus, and Web of science was carried out.

The search was performed using the following keywords: the Pcrit, pharyngeal collapsibility, sleep apnea and/or pressure, collapsibility of upper airway, collapsibility and/or OSA, sleep-disordered breathing and/or collapsibility, pressure and/or upper airway, and pharyngeal pressure. The references in the articles that were retrieved, were also reviewed to identify additional relevant articles. Furthermore, the cited and related citation features of the databases were utilized to identify additional articles of importance.

No limitations were considered in the format of the included studies and they could be in the form of posters, abstracts, original research, case reports, case series, literature reviews, systematic reviews, and meta-analyses (written in English). Elahe Kazemeini then reviewed all the relevant articles, to extract the information.

## Results

In the following sections, the physiologic models used to explain the pharynx and Pcrit, such as the Starling resistor model, the Pcrit definition, its measurement methods, the physiologic factors that affect Pcrit, and the effect of Pcrit on various therapies will be explored.

### Starling Resistor

**Figure 1:**
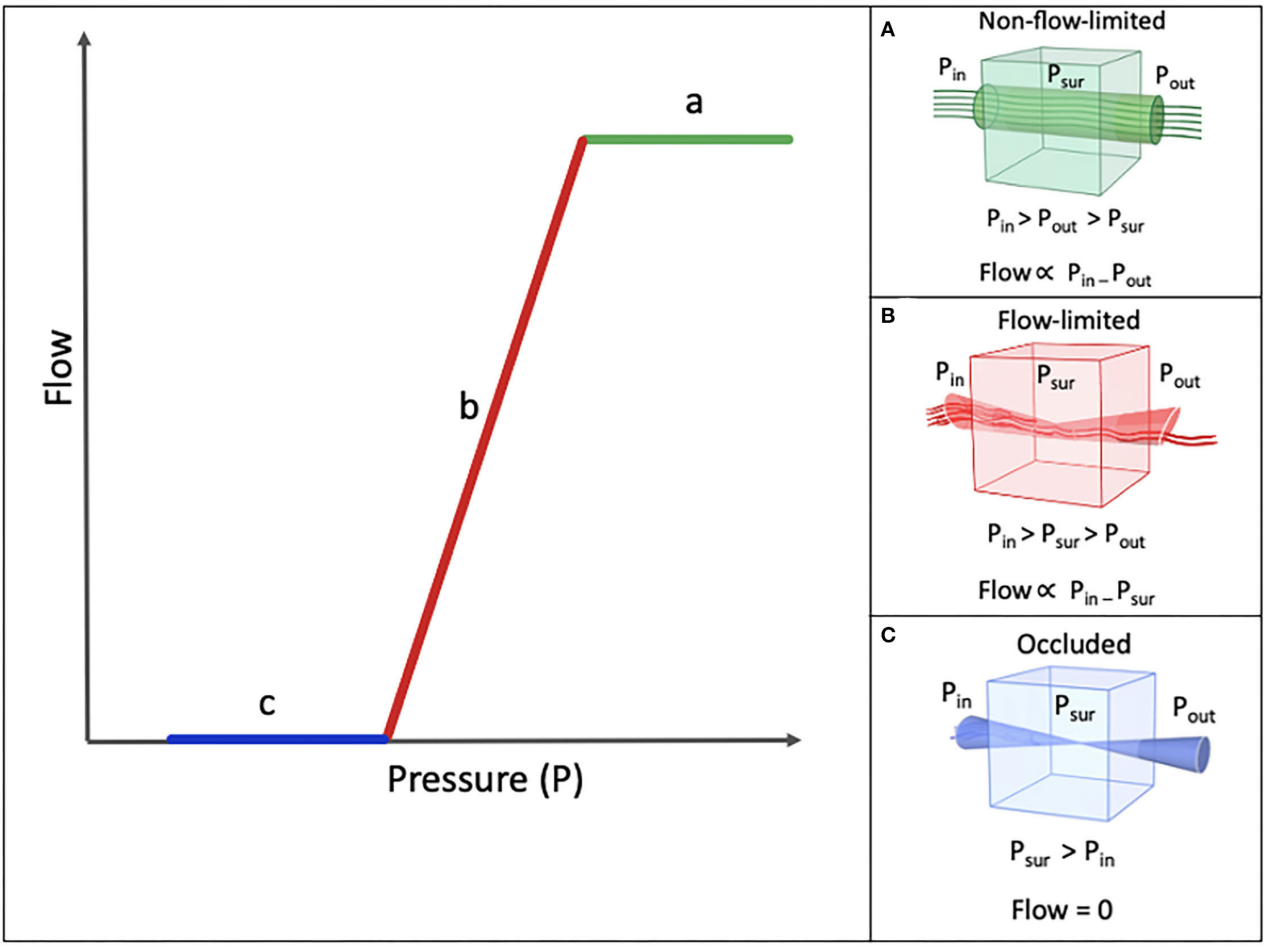
Schematic model of Pressure-Flow relationship. P_in_ (inflow pressure), P_out_ (Outflow pressure), and P_sur_ (surrounding pressure). **(A)** Nonflow-limited segment. **(B)** Flow-limited segment. **(C)** Occluded segmet.

Knowlton and Starling devised a model for collapsible tube behavior (9). This Starling resistor model is often used to describe various functions of most of the biologic systems, such as the vascular system, respiratory system, as well as the pharyngeal airway, whose so-called collapsible segment has no rigid support throughout most of its trajectory, except for its proximal (posterior aspect of the nasal septum) and distal (larynx) ends, which makes it prone to collapse. Knowlton and Starling used a device that was made of a thin-walled collapsible tube traversing a chamber, by means of which they could control the peripheral resistance. In this device, the pressure of the surrounding chamber could be set to any given value (9). Green et al. (10) summarized the modalities of an ideal Starling resistor in three statements (Figure 1):

A. When the inflow pressure is greater than the outflow pressure, and the outflow pressure is greater than the surrounding pressure, the flow is proportional to the difference between the inflow and the outflow pressure, independent of the surrounding pressure (Figure 1A) (10).
B. When the inflow pressure is greater than the surrounding pressure, and the surrounding pressure is greater than the outflow pressure, the flow is proportional to the difference between the inflow pressure and the surrounding pressure, independent of the changes in the outflow pressure (Figure 1B) (10).
C. When the surrounding pressure is greater than the inflow pressure, there will be no flow throughout the tube (Figure 1C) (10).

The maximal flow that can be achieved is determined by the properties of the collapsible segment. According to the Starling resistor, the pressure gradient that drives the flow and the maximum flow level is fixed. Based on Poiseuille's law, three factors determine the flow through a tube: the length of the tube, the viscosity of the flowing material, and the diameter of the passage. Since under normal physiologic conditions, the length and the viscosity of the upper airway remain constant, the only factor that affects the resistance to the flow will be the upper airway diameter (Figure 1B) (9, 11).

### Definition of Pcrit

Pharyngeal Pcrit is the pressure at which the airway can no longer stay open and collapses (10, 12). It is a measure of the level of collapsibility of the pharyngeal airway. As discussed, the pharynx can be modeed as a Starling resistor. Any increase in the surrounding pressure higher than the internal pressure within the tube, results in the collapse of the pharynx. At the moment of collapse, right before the surrounding pressure rises to a level above the pressure inside the tube and causes its collapse, the pressure within the tube is equal to the surrounding pressure. The pressure at this specific moment is known as the Pcrit of that segment (8). Any increase in the surrounding pressure above this critical value, results in the collapse of the tube (Figure 1). The more negative the pressure is, compared to the atmospheric pressure, less effort is needed to open the airway. Therefore, the lower the Pcrit is, the less collapsible the upper airway is.

Findings of Butler et al., Wellman et al., Owens et al., and Genta et al. contradict the Starling resistor model of Pcrit (13–16). During the flow-limited phase of the Starling resistor model (Figure 1B), Pcrit is the main determinant of flow. However, these recent studies observed a negative effort dependence during inspiration in the flow-limited phase, meaning that the flow was decreased despite an increase in the inspiratory effort (13–16). Negative effort dependence is defined as the percentage of reduction in the inspiratory flow from the peak to the plateau. Breaths with high negative effort dependence have a high peak at the start of inspiration, followed by a low plateau inspiration (11–14). Based on these studies, we can conclude that the Starling resistor model is only valid in patients with low negative effort dependence. These results show that not one model fits all subjects with OSA and multiple models can be applied for different clusters of patients.

### Measurement of Pcrit

#### Types

There are two types of measured Pcrit (**Figure 5**):

- Passive Pcrit, the Pcrit caused while the effect of the muscles is eliminated by the measurement method. This is an estimate of the airway pressure and behavior under passive mechanical loading.
- Active Pcrit, the Pcrit caused while taking into account, the contribution of the pharyngeal muscle activation. It is an estimate of the pressure and behavior of the airway with active neuromuscular response.

Both the types of Pcrit require a slightly different measurement technique, yet can be measured using the same set-up.

#### Pcrit Measurement Set-Up

**Figure 2:**
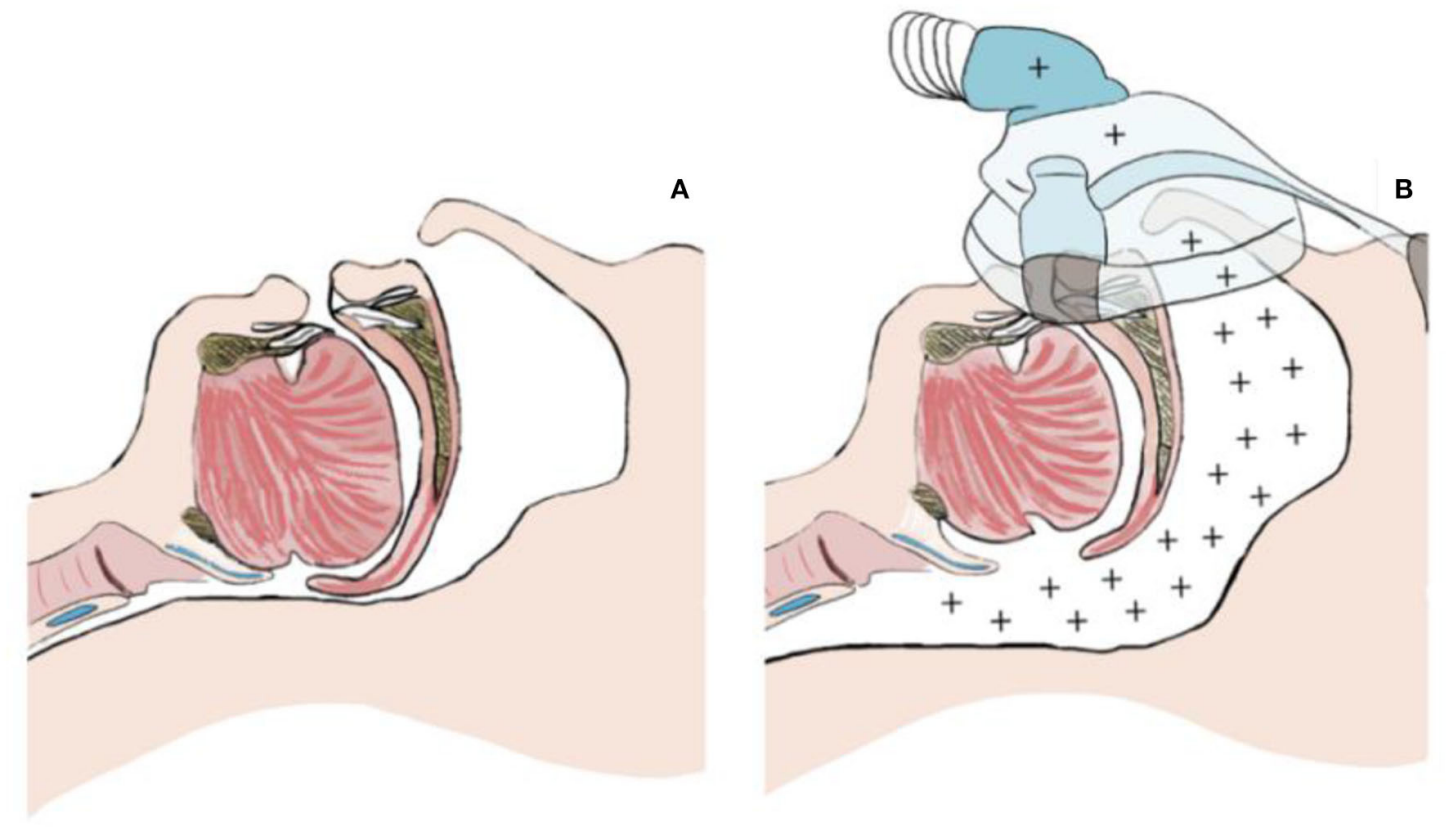
**(A)** Collapsed airway. **(B)** Open airway with a continuous positive airway pressure (CPAP).

Pcrit measurement techniques are based on continuous positive airway pressure (CPAP) treatment principles, which is the standard non-invasive treatment of OSA. The CPAP creates a pneumatic splint against the collapse of the structures of the pharyngeal airway by blowing the positive pressured air into the airway to keep its structures open (Figure 2) (17).

**Figure 3:**
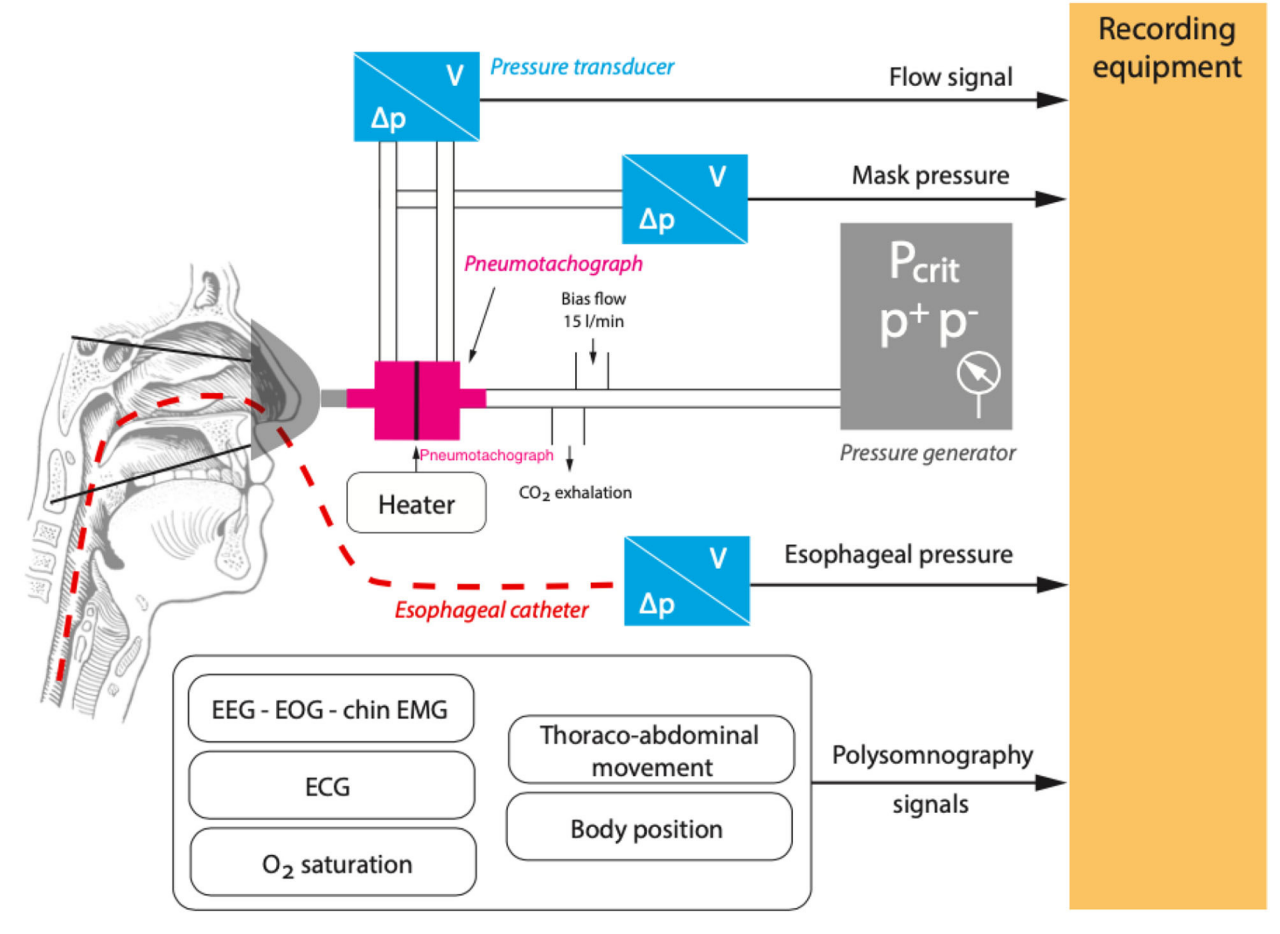
A generic set-up for the measurement of critical closing pressure (Pcrit). Airflow (V), pressure difference (ΔP), electrocardiography (ECG), electroencephalography (EEG), electromyography (EMG), Oxygen (O_2_), positive pressure (P^+^), and negative pressure (P^−^).

To measure the Pcrit, a generic set-up has been developed (Figure 3) (12, 18–21). The aim of the study is to supinely position, the subject, irrespective of the effect of the other positions on Pcrit. A full polysomnography (PSG) set-up is used to record various parameters. These include sleep stages through electroencephalography (EEG), surface muscular activity using electromyography (EMG) of the chin and legs, electrocardiography (ECG), and thoracic and abdominal inductive plethysmography bands to record the respiratory effort and oxygen saturation using an oximeter. To measure the airflow, a nasal mask attached to a heated pneumotachometer and a differential pressure transducer is worn by the subject. The flow and the pressure of the mask is recorded on a computer. Special effort should be put in avoiding air leaks. An esophageal pressure catheter is inserted through one of the nostrils for the measurement of the respiratory effort. A modified CPAP device capable of producing both negative and positive pressures is used. This set-up is a closed-loop. Therefore, a whisper swivel to prevent rebreathing and a bias flow of oxygen for periods of zero CPAP flow is added to the set-up after the pneumotachometer and before the CPAP device (12, 18–20).

#### Gold Standard Pcrit Measurement Techniques

**Figure 4:**
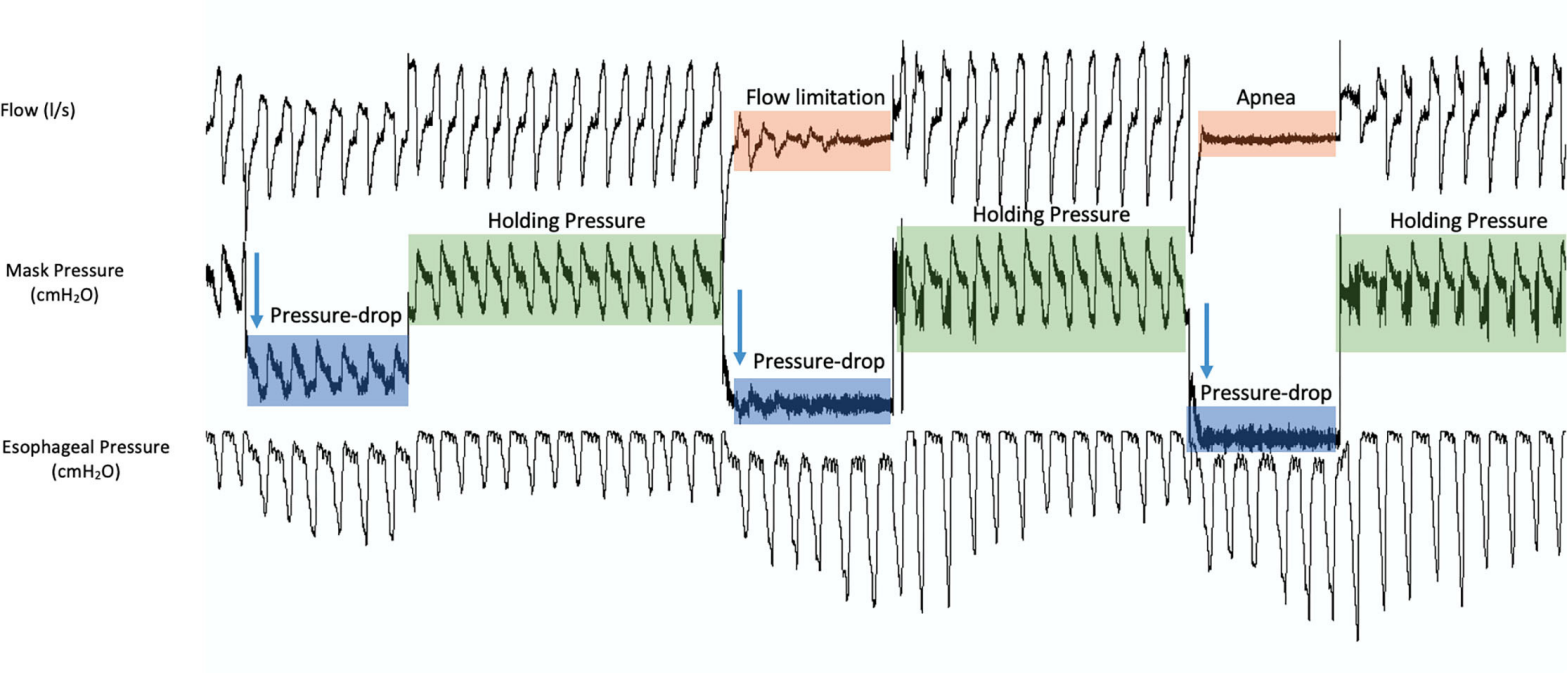
A sample of 3 pressure drop series during the measurement of critical closing pressure (Pcrit). During measurements, the pressure is increased to a level where the pharyngeal airway is open and the airflow is not limited (holding pressure, green). Then, multiple series of pressure drops are performed. Starting at the holding pressure, the pressure is reduced to a lower pressure for a set number of breaths (blue), inducing flow limitation (orange), and then back to the holding pressure. This process is repeated until a pressure at which the airflow is zero (apnea, orange).

Various methods for Pcrit measurement have been introduced. The core of most of these methods is to increase the pressure to a level where the pharyngeal airway is open and the airflow is not limited (Figure 1A), called the “holding pressure.” Then, multiple series of pressure drop (Figures 1B, 4) will be performed to obtain a pressure at which the airflow is zero (Figure 1C). Thereafter, the pressureflow relationship is calculated using the linear regression to determine the Pcrit (Figure 1).

Some methods use the peak inspiratory flow of the recorded breath signals as a measure of flow, while others use the inspiratory ventilation for Pcrit calculations (12, 22–24).

**Figure 5:**
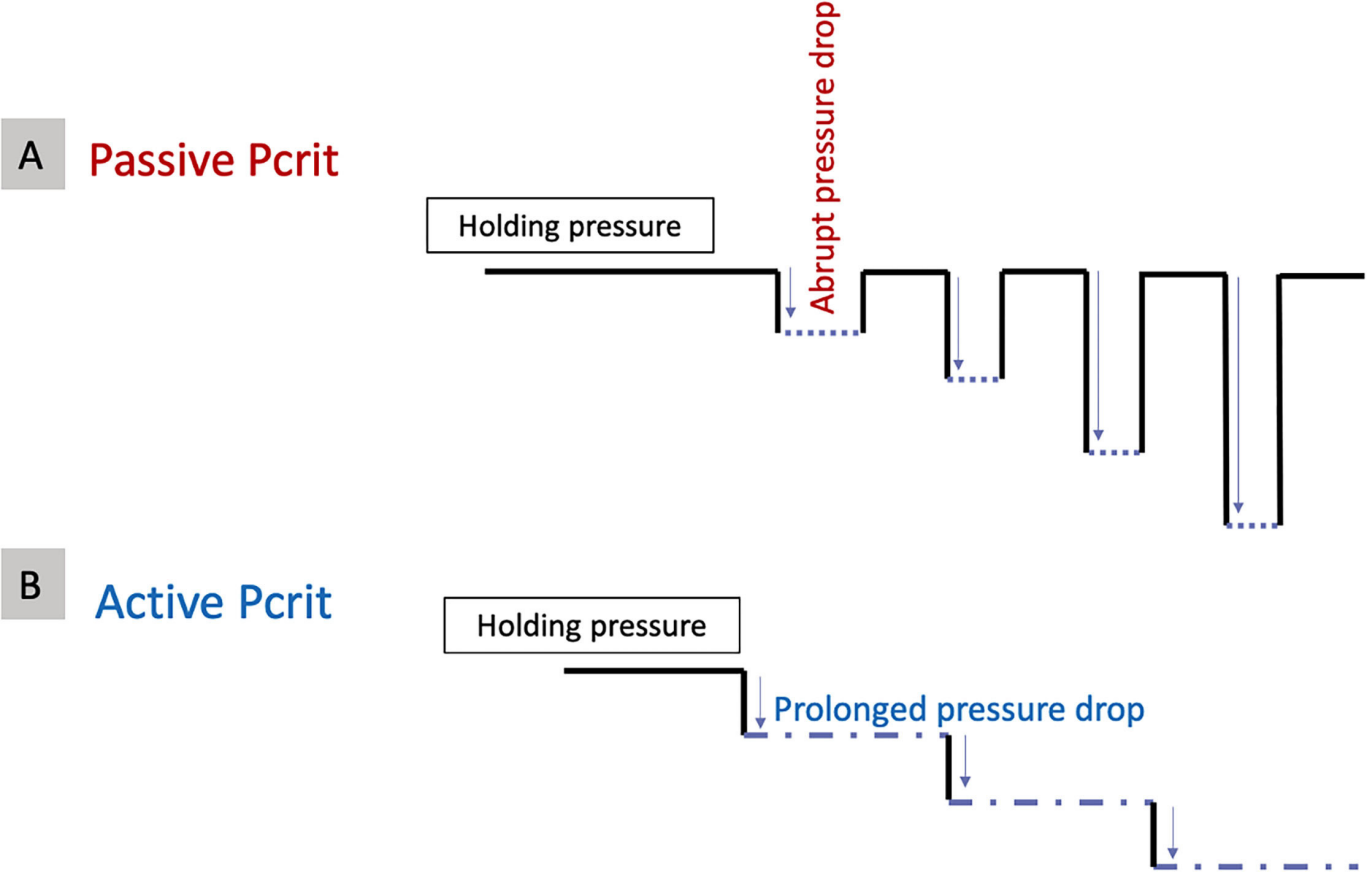
Different methods to measure **(A)** Passive critical closing pressure (Pcrit) and **(B)** Active Pcrit.

As mentioned, two types of Pcrit can be measured. During these pressure measurements, if the pressure is dropped abruptly, the measured critical closing pressure is called, the “passive Pcrit.” Passive Pcrit is measured in this way so that the airflow is recorded before the activation of the dynamic airway muscle activation (25). After finding the “holding pressure,” multiple series of pressure drops will be performed in specific pressure increments (for example, increments of 1–2 cmH_2_O for five breaths each) until a pressure at which the airflow is zero. Between each pressure drop, the pressure will be set back to the holding pressure (Figure 5A). The “active Pcrit” is measured by a slow and progressive reduction of the pressure as depicted in Figure 5B. This method allocates enough time for dilator muscles of the upper airway to activate (12, 18–20, 25). In this method, the pressure is dropped from the holding pressure for a longer period of time (10 min) and there will be no returning to the holding pressure between each pressure drop (Figure 5B).

#### Evolution of Pcrit Measurements

For the first time in 1984, Issa and Sulivan measured the active Pcrit in 18 subjects with OSA (12). Schwartz et al. optimized this method in healthy volunteers (22). Originally, the recording was carried out by setting a holding pressure of −1 cmH_2_O for all the subjects. Subsequently, the pressure drop of 3–5 min, allowing for the upper airway muscles to be recruited, was performed throughout the whole duration of the night until the subject was unable to fall back asleep again (22).

To unify the Pcrit measurements, an “abbreviated method” was introduced (23). First, a personalized holding pressure was determined. Subsequently, a pressure drop was performed for the duration of five breaths with 1 min at the holding pressure in between each drop, instead of the long 3-to-5-min pressure drop of prior measurement methods (23). In the abbreviated method, all breaths of the pressure drop period were analyzed to establish whether they were flow-limited or not (23).

In recent studies, 3–5 breaths from a 5-breath pressure drop is used for further analysis (23, 26, 27). During a pressure drop, usually it takes two breaths after the change of the pressure to reach a steady air flow state. This might be due to the peak inspiratory flow adjustments to the end-expiratory lung volume or by the increasing respiratory effort due to arousal stimuli (26–28).

To further facilitate Pcrit calculation, a “simple method” was proposed (29). In this method, first, all breaths were plotted against their associated pressures, to find the flow-limited segment. On this pressure-flow plot, an upper inflection point (between portions a and b on Figure 1) and a lower inflection point were identified (between portions b and c on Figure 1). Hereafter, the range of pressure between these two inflection points, over which the flow is markedly varied (Figure 1B), is isolated. This range is used for calculating Pcrit (29).

#### Alternative Methods for Pcrit Measurement

Pcrit measurements using the gold standard method are performed during natural sleep. However, this is cumbersome and labor-intensive and therefore not feasible for routine practice. To overcome the problem of recurring arousals while measuring Pcrit during natural sleep and reduce the time needed to measure the upper airway collapsibility, other methods were investigated.

##### Pcrit Measurement During Anesthesia

Measurement of Pcrit during drug-induced sleep with midazolam (−0.97 ± 3.21 cmH_2_O showed to produce a Pcrit comparable to that of natural sleep (−0.82 ± 3.44 cmH_2_O) (18). However, application of different concentrations of propofol (effect-site concentration = 2.5, 4.0, and 6.0 μg/ml) showed that deeper anesthesia increases the collapsibility (−0.3 ± 3.5, 0.5 ± 3.7, and 1.4 ± 3.5 cmH_2_O at propofol concentrations of 2.5, 4.0, and 6.0 μg/ml respectively; *P* < 0.05) (30). Step-wise induction of anesthesia with propofol (0–3 μg/ml) showed a non-linear relationship between the loss of consciousness and elevation of collapsibility (31). This increase in collapsibility during anesthesia might be related to the loss in consciousness causing the genioglossus muscle activity to decrease abruptly while transitioning from awake to sedation state (31). Passive Pcrit is associated with the tonic activity of pharyngeal dilator muscles including the genioglossus muscle (32, 33). Increasing the baseline tonic dilator muscle activity reduces the collapsibility, which in turn increases the stiffness of the upper airway (33–35).

##### Collapsibility Measurements During Awake State

Furthermore, several studies were performed to evaluate awake parameters as a surrogate for Pcrit (36–39). The upper airway collapsibility index is measured by the application of negative pressure pulses of −12 cmH_2_O in brief periods (250 ms) every 8–10 breaths intermittently at the early inspiration. The upper airway collapsibility index showed to have a strong association with Pcrit and therefore might be a viable marker of the pharyngeal collapsibility level (36, 37).

A previously developed awake method used a negative expiratory pressure maneuver (38). The patient was positioned supine while wearing a nasal mask connected to a pneumotachometer. During each maneuver, a software-operated negative pressure producing device applied negative pressure to the pharyngeal airway for the duration of 2 s. Ten negative expiratory pressure maneuvers were performed in each subject with at least four regular breaths in between. The ratio of mean exhaled volume at 0.2 s during stable breathing and the exhaled volume at 0.2 s during negative expiratory pressure were used to estimate the surrogate pressure (38). However, this method showed a weak correlation with Pcrit (*r* = 0.3) (38).

##### Collapsibility Measurements Using Computational Modeling

Azarbarzin et al. have proposed the possibility of the estimation of the level of collapsibility *via* air flow measurements without the need for time-consuming Pcrit measurement methods (24). The main hypothesis is that maximum airflow at atmospheric pressure reflects the Pcrit. This implies that the slope of the regression lines in a pressure-flow curve is similar at the atmospheric pressure compared to at CPAP pressures and it can therefore be used to extrapolate the Pcrit level. This team performed peak and mid-inspiratory flow measurements during the first and third half of the night, off CPAP. In the mid part of the night, they connected the modified CPAP device to a nasal mask worn by the patient and estimated active and passive Pcrit (Passive Pcrit = −0.7 cmH_2_O, VpeakNREM *r* = −0.007 and active Pcrit = −3.5 cmH_2_O, VpeakNREM *r* = −0.71) (24).

Furthermore, Sands et al. developed a technique to determine the collapsibility of the upper airway from clinical polysomnography using a non-invasive computational modeling technique, based on the simplified method (27, 40, 41). In this method, the passive collapsibility is quantified by the median ventilation at the eupneic ventilatory drive, while active collapsibility is defined as the median ventilation at the maximal ventilatory drive (at the arousal threshold). The higher these ventilation values, the lower is the collapsibility and vice versa. The results from these models were strongly associated with gold standard Pcrit measurement techniques (Passive *r* = 0.67 *p* < 0.0001, active *r* = 0.81 *p* < 0.0001) (27, 40, 41).

The finite element (FE) modeling is an emerging technique. These are computational models that give information on partial differential equations of problems of time and space in physics. Using imaging techniques (CT and MRI) and special software, models of pharyngeal soft tissue are reproduced. These models have been validated for the human pharyngeal airway (42), and employing them helps to better understand the anatomical and physiological behavior of the pharyngeal airway. Pcrit and the changes related to it under different loads were evaluated with this model (16, 43–46).

The cross-sectional area of the upper airway has been used to develop a mathematical model of the pressure/area of the upper airway which can predict the upper airway collapsibility. To have an estimate of this cross-sectional area, one might use an upper airway endoscopy, an MRI, or acoustic pharyngometry (47–53). In 247 children, the cross-sectional area of the airway was measured before and after administering 1% of cetacaine with an acoustic pharyngometry. The segment at which the percentage of pre- and postcetacaine difference was maximal was further analyzed and used to define the upper airway collapsibility in subjects (52). An upper airway collapsibility of ≤ -30% was highly sensitive and specific in the identification of subjects with OSA and decreased in second or third after tonsillectomy (52).

#### Pcrit Values

The normal adult pharyngeal airway has a negative Pcrit (−13.3 ± 3.2 cmH_2_O) during sleep and by lowering the nasal pressure below this Pcrit, occlusion is induced (22). The more positive the Pcrit is, the more collapsible the pharynx is. The Pcrit of primary snorers is estimated to be around −6.5 ± 2.7 cmH_2_O. In patients with OSA, more positive values are present, approximately around 2.5 ± 1 cmH_2_O and almost never below −5 cmH_2_O (3). Table 1 gives an overview of the measured Pcrit from various studies (19, 22, 54, 60, 61).

**Table 1:** Overview of the measured critical closing pressure (Pcrit) from various studies under various situations with varying subject status.

**Subject status**	**Pcrit (cm of H_**2**_O)**	**AHI (events/hour)**	**BMI (kg/m^**2**^)**	***n***	**References**
**Pcrit in healthy subjects during various body positions**					
Healthy subjects	−13.3 ±3.2	NA	24.2 ± 1.9 (males) 19.7 ± 0.8 (females)	7	(22)
Healthy, on side asleep	4.8 ± 0.5	NA	23.3 ± 0.5	38	(36)
Healthy, on side awake	2.5 ±2.5	NA	23.3 ± 0.5	38	(36)
Healthy	−15.4 ± 6.1	NA	24	12	(54)
Healthy, Neutral neck position	−0.4 ± 4.4	NA	23.8 ± 2.4	15	(55)
Healthy, Neck flexion	3.7 ± 2.9	NA	23.8 ± 2.4	15	(55)
Healthy, Neck extension	−9.4 ± 3.8	NA	23.8 ± 2.4	15	(55)
Healthy, Neck rotation	−2.6 ± 3.3	NA	23.8 ± 2.4	15	(55)
Healthy, Neutral neck position	−0.4 ± 4.4	NA	23.8 ± 2.4	15	(55)
Healthy, Neck flexion	3.7 ± 2.9	NA	23.8 ± 2.4	15	(55)
Healthy, Neck extension	−9.4 ± 3.8	NA	23.8 ± 2.4	15	(55)
Healthy, Neck rotation	−2.6 ± 3.3	NA	23.8 ± 2.4	15	(55)
**Pcrit in OSA subjects, primary snorers and obstructive hypopnea during various body positions**					
OSA	2.5 ± 1.5	67.6 ±23.9 (RDI)	29.3 ± 3.2	10	(19)
OSA	5.6 ± 0.5	63 ± 7	38 ± 2.1	22	(36)
OSA	2.4 ± 2.8	> 40	38	47	(54)
OSA	−1.6 ± 2.6	10–40	33	37	(54)
OSA	−0.98 ± 2.47	68.3 ± 27.0 (NREM AHI)	36.6 ± 6.7	30	(29)
OSA	−0.7 (passive Pcrit)	29.8	30.4	14	(24)
OSA	−3.5 (active Pcrit)	29.8	30.4	14	(24)
OSA	−0.11 ± 2.5	34 ± 23	31 ± 6	34	(37)
OSA Neutral neck position retropalatal	2.68	55.1 (ODI)	28.3	12	(56)
OSA Sniffing neck position retropalatal	−1.78	55.1 (ODI)	28.3	12	(56)
OSA Neutral neck position retroglossal	0.80	55.1 (ODI)	28.3	12	(56)
OSA Neutral neck position retroglossal	−3.72	55.1 (ODI)	28.3	12	(56)
OSA, supine body position	1.8	63.0 ± 14.6 (RDI)	32 ± 5.6	10	(23)
OSA, lateral body position	−1.1	63.0 ± 14.6 (RDI)	32 ± 5.6	10	(23)
OSA, Supine position	2.02 ± 2.55	57 ± 23.8	34.7 ± 6.3	20	(57)
OSA, Lateral position	−1.92 ± 3.87	57 ± 23.8	34.7 ± 6.3	20	(57)
Primary snorer	−6.5 ± 2.7	1 ± 1.4 (RDI)	30.9 ± 4.2	10	(19)
Obstructive hypopnea patients	−1.6 ± 1.4	48.1 ± 17.6 (RDI)	34.5 ± 5.1	6	(19)
**Pcrit in OSA during various sleep stages**					
OSA, N1 and N2 sleep stages	3.1 ± 0.4	55 ± 4.4 (apneic index)	_	18	(12)
OSA, N3 and N4 sleep stages	4.2 ± 0.2	55 ± 4.4 (apneic index)	_	18	(12)
OSA, REM sleep	2.4 ± 0.2	55 ± 4.4 (apneic index)	_	18	(12)
OSA, N2	0.74 ± 0.03	36 ± 4	44 ± 1.4	33	(58)
OSA, REM	0.84 ± 0.03	36 ± 4	44 ± 1.4	20	(58)
**Pcrit in natural sleep vs. drug–induced sleep**					
OSA, natural sleep	−0.82 ± 3.44	38 ± 22	30 ± 4	15	(18)
OSA, midazolam induced sleep	−0.97 ± 3.21	38 ± 22	30 ± 4	15	(18)
6.0 μg/ml propofol	1.4 ± 3.5	NA	28 ± 3	12	(30)
4.0 μg/ml propofol	0.5 ± 3.7	NA	28 ± 3	12	(30)
2.5 μg/ml propofol	−0.3 ± 3.5	NA	28 ± 3	12	(30)
Isoflurane 1.2%	± 3.5	NA	28.3 ± 4.3	16	(59)
Isoflurane 0.4%	−0.2 ± 3.6	NA	28.3 ± 4.3	16	(59)

*AHI, Apnea-hypopnea index; BMI, body mass index; n, number of subjects included in the relevant study, reference: the study from which the data was extracted, NA, not applicable; NREM AHI, non-rapid eye movement sleep AHI; ODI, oxygen desaturation index; RDI, respiratory disturbance index; REM, rapid eye movement sleep; OSA, obstructive sleep apnea*.

Healthy subjects are able to decrease their active Pcrit (−11.1 cmH_2_O) to a lower level (−4.5 cmH_2_O) after passive loading of the upper airway. However, OSA subjects seem to be unable to lower the Pcrit after loading (from active Pcrit = −0.05 cmH_2_O to passive Pcrit = −1.6 cmH_2_O) (62).

### Determinants of Pharyngeal Collapsibility

Various physiologic parameters, such as anthropometric features, the body or neck position, gender, age, ethnicity, sleep quality and sleep stage, and the respiratory cycle itself affect Pcrit.

#### Anatomy and Body Composition

Obesity [body mass index (BMI) ≥ 30 kg/m^2^], an increase in the proportion of the soft tissue volume to the surrounding bone, enlarged lateral pharyngeal walls and soft palate, and an inferiorly located hyoid bone (increased pharyngeal length as measured by mandibular-hyoid plane distance) increase the pharyngeal collapsibility (60, 63–65).

#### Body or Neck Position

Neck flexion decreases the pharyngeal patency and therefore increases the collapsibility. Pcrit increases during neck flexion regardless of the body position in supine, prone, or while turning the head to the side (66). In contrast, the sniffing neck position (8 cm elevation) increases the cross-sectional area of the pharynx and results in a Pcrit decrease at the retropalatal and retroglossal levels (retropalatal: 2.68 to −1.78 cmH_2_O, retroglossal: 0.80 to −3.72 cmH_2_O) (56). In 15 healthy individuals, Pcrit in the neutral neck position was −0.4 ± 4.4 cmH_2_O. It increased to 3.7 ± 2.9 cmH_2_O by neck flexion, decreased to −9.4 ± 3.8 cmH_2_O by extension and remained unchanged by rotation (−2.6 ± 3.3 cmH_2_O). These measurements clearly indicate that head posture might have a compelling effect on the pharyngeal collapsibility (55).

Body position during sleep affects the Pcrit as well. Changing the position from supine to lateral decreases the collapsibility (23, 57). Boudewyns et al. (23) compared the effect of supine vs. lateral body position on Pcrit values in 10 patients with obesity having a respiratory disturbance index (RDI) equal to 63.0 ± 14.6/h. Pcrit in the supine position was 1.8 and plummeted to −1.1 cmH_2_O by changing the position to lateral during the measurements. However, no significant position dependency was observed in the upstream resistance values (23). Furthermore, in a different study, the lateral position significantly reduced the collapsibility from 2.02 ± 2.55 to −1.92 ± 3.87 cmH_2_O, in 20 patients with OSA (57).

#### Gender

Pharyngeal resistance and collapsibility between men and women is not significantly different. The mean Pcrit in men and women without OSA was −10.4 ± 3.1 and −8.8 ± 2.7 cmH_2_O, respectively (67). The role of gender was investigated in two groups: the first one consisting of 11 men and 11 women matched for the severity of OSA (AHI: 43.8 ± 6.1 and 44.1 ± 6.6 events/h) and the second one including 12 men and 12 women matched for BMI (31.6 ± 1.9 and 31.3 ± 1.8 kg/m^2^).

In the OSA-severity matched group; women had a higher BMI. Yet, no difference among genders were found for the collapsibility (0.35 ± 0.62 vs, −0.18 ± 0.87 cmH_2_O, *P* = 0.63). In the BMI-matched group; women had less severe OSA and a lower Pcrit compared to men (−2.01 ± 0.62 vs. 1.16 ± 0.83 cmH_2_O; *P* = 0.005). These data suggest that the effect of being overweight/obese on OSA might be greater in men compared to women and hence women have a less collapsible pharynx for any given BMI as in the male who have the same BMI (68). A recreated finite element model of the male and female pharyngeal airway confirmed this finding (69). This might be due to the different anatomical characteristics, such as a longer pharyngeal airway, an increased cross-sectional area of the soft palate, and increased airway volume in men compared to women (69).

#### Age

##### Pediatric Population

During infancy, Pcrit (−0.5 and −0.7 ± 2 cmH_2_O) is close to atmospheric pressure which is a high risk for collapse (66, 70). After the 1st year of life, the upper airway muscles become more active resulting in a more stable and less collapsible airway (Pcrit = −6 cmH_2_O) (70).

Compared to adults, children have a less collapsible airway (71). During sleep, children have an active upper airway dynamic neuromotor response to negative pressures which keeps the airway open (71, 72). The upper airway is very resistant to collapse in healthy children and Pcrit can hardly be measured with the gold standard measures of Pcrit assessment (73). Still, comparing healthy and OSA children show a more collapsible airway with an elevated airway resistance in the OSA group (47, 74–76). Furthermore, the passive pharynx of children with OSA is more vulnerable to the changes in pressure near closing pressures, independent of the size of the pharynx (47).

The upper airway reflexes which keep the airway open are reduced during adolescence (77). Compared to the healthy obese adolescents with OSA, obese adolescents showed that healthy subjects preserve this protective reflex into adolescence while the subjects with OSA lose this protective measure and cannot keep the airway open during sleep (77).

##### Adults and Geriatric Population

As mentioned, the normal adult airway in healthy subjects has a more negative Pcrit which increases to more positive values in patients with OSA. Aging, regardless of gender, slightly increases the collapsibility and makes the airway more prone to collapse (by 0.6 cmH_2_O per decade) (78). Aging also results in an elevated deposition of parapharyngeal fat, regardless of baseline BMI, and the lengthening of the soft palate (especially in women) which are all associated with higher collapsibility (69, 79, 80). The upper airway neuromotor response to negative pressures also decreases with aging (81, 82). All these parameters can contribute to a higher prevalence of OSA with aging (83, 84).

#### Ethnicity

Ethnicity and genetics affect the shape and structure of the pharyngeal airway. As an example, a comparison of Pcrit between the Whites and the Japanese Brazilians showed no statistically significant difference. However, the craniofacial characteristics that affect the Pcrit were different between the two groups. In the Whites, the collapsibility was correlated with the ratio of the tongue volume to the mandible volume. In the Japanese Brazilians, it was correlated with the cranial base angle (85).

#### Sleep-Wake State

The upper airway is more prone to collapse during sleep (3). During both the awake and sleep state, the human airway has compensatory mechanisms, such as neuromuscular reflexes to the negative pressure, to stay open. These compensatory reflexes are reduced during sleep. In subjects with OSA, this loss is more prominent during sleep compared to the healthy subjects. Therefore, as patients with OSA rely mainly upon these neuromuscular reflexes to keep the airway open, this will result in a more collapsible upper airway (33, 86, 87). Collapsibility during sleep, in healthy controls in the lateral posture was significantly higher than during the awake state (54.4 vs. 27.5%; *p* < 0.01) (34).

#### Stage and Quality of Sleep

Sleep stage and the quality of sleep may affect Pcrit. According to one study, no difference between Pcrit during sleep stages N1/N2 (3.1 ± 0.4) and rapid eye movement (REM) sleep (2.4 ± 0.2 cmH_2_O) was found (12). However, there was a significant difference between the collapsibility in other sleep stages and that of slow-wave sleep (4.2 ± 0.2 cmH_2_O), showing a higher collapsibility during the deeper sleep (12). Contradictory to this, another study found that Pcrit during REM was 4.9 ± 1.4 cmH_2_O higher than that of deeper sleep stages, implying a more collapsible upper airway during REM sleep (58).

Sleep deprivation (one night of total sleep deprivation prior to measurements) and sleep fragmentation (using auditory stimuli) in subjects with OSA resulted in a more collapsible airway (12.3 ± 6.3 cmH_2_O). However, sleep deprivation in healthy subjects had no effect on collapsibility (−17.1 ± 6.8 cmH_2_O) (88).

#### Respiratory Cycle

Within a single respiratory cycle, the collapsibility and upstream resistance varies between inspiration and expiration, with Pcrit during expiration being up to 4 cmH_2_O more than during inspiration. This variation might be due to both local mechanical and neuromuscular factors (89).

### Treatments and Pcrit

In the phenotypic models of subjects with OSA, it has been shown that subjects with a Pcrit ≥ +2 cmH_2_O respond better to major anatomic and mechanical interventions, such as CPAP, while those with a Pcrit ≤ +2 cmH_2_O might benefit from other therapies, such as mandibular advancement device (MAD), surgical interventions, medications, or a combination of these targeted therapies (7).

#### Continuous Positive Airway Pressure

Therapeutic CPAP levels show a strong relationship with collapsibility. The needed CPAP level, to resolve OSA, is lower in patients with a milder Pcrit (≤ -2 cmH_2_O) compared to a higher Pcrit (>-2 cmH_2_O) collapsibility. Therefore, the level of therapeutic CPAP might be indicative of the level of collapsibility (90). The CPAP did not affect Pcrit during N1/N2 sleep stages. However, during deep sleep, for every 1 cmH_2_O rise in CPAP pressure, a 0.65 cmH_2_O decrease in the Pcrit was shown (12).

#### Weight Loss

Weight loss decreases collapsibility. In 23 (BMI: 42 ± 7 kg/m^2^) patients with obesity, severe OSA (disordered breathing rate: 83.3 ± 31/h), and high collapsibility (Pcrit: 3.1 ± 4.2 cmH_2_O), the effect of weight loss was evaluated (91). Thirteen patients reached the goal of 5% reduction in body weight and were included in the final evaluations. After 16.9 ± 10.0 months of follow-up, the disordered breathing rate decreased to 32.5 ± 35.9/h and the Pcrit to −2.4 ± 4.4 cmH_2_O. The reduction in collapsibility was significantly correlated with the reduction in BMI. A decrease of Pcrit to below −4 cmH_2_O was associated with a near-complete elimination of OSA (disordered breathing rate <20/h) (91).

#### Mandibular Advancement Device

Mandibular advancement devices protrude the mandible to open up the airway (92). This forward positioning of the mandible affects the pharyngeal collapsibility and decreases the Pcrit (93–95). The MAD therapy significantly increases both the passive ventilation (*p* = 0.002) and active ventilation (*P* < 0.001) (95). Pcrit in four different positions was measured. Neutral (−4.2 ± 2.9 cmH_2_O) centric occlusal meaning zero mm advancement (−7.1 ± 5.2 cmH_2_O), incisors aligned (−10.7 ± 4.4 cmH_2_O), and 75% of maximal advancement of the mandible (−13.3 ± 3.2 cmH_2_O). Mandibular protrusion to the “incisors aligned” position significantly decreased the collapsibility. Further advancement toward the maximal position reduced the Pcrit even more (96). These results were also confirmed by another study. Ayuse et al. showed that the advancement of the mandible from centric occlusion to maximal protrusion by 7.1 ± 1.2 mm, significantly reduces Pcrit (from 1.9 ± 2.9 cmH_2_O to −7.3 ± 1.9 cmH_2_O) (93). It has been demonstrated that the effect of mandibular advancement is dose-dependent and works through improving the passive pharyngeal airway rather than affecting the function of the genioglossus muscle (94). In 12 patients, the measurement of collapsibility in three different positions of neutral, 50%, and 100% of maximum comfortable mandibular advancement clearly manifested this stepwise dose-dependent effect of mandibular advancement in reducing the collapsibility with a decrease in Pcrit from 1.8 ± 3.9 cmH_2_O to −0.9 ± 2.9 cmH_2_O, and −4.0 ± 3.6 cmH_2_O, respectively (94).

A less collapsible airway is an independent predictor of response to MAD, defined as a percent reduction in AHI (non-linear multivariate model, *r*^2^ = 0.70) (95). Non-responders to MAD therapy were shown to have a higher baseline collapsibility (bivariate model, p = 0.014) (97). This shows the importance of Pcrit not only in the follow-up of MAD therapy and efficacy evaluations, but also in the prediction of treatment success and the choice of the therapy option (95, 98).

#### Anatomic Manipulations—Upper Airway Surgery

Using FE modeling techniques, the effect of three different therapy options on collapsibility was investigated. The benefit of using such a model is that it allows for comparing all manipulations in the same individual. The baseline Pcrit was −13 cmH_2_O. All three treatment modalities, such as mandibular advancement (−21 cmH_2_O), palatal resection (−18 cmH_2_O), and palatal stiffening by palatal implants (−17 cmH_2_O) decreased the collapsibility (99). In another model with baseline Pcrit of −2 cmH_2_O, uvulopalatopharyngoplasty (UPPP) reduced the collapsibility with tissue resection to a certain sufficient level. However, after sufficient palatal resection, Pcrit was improved. However, with further palatal resection, retro-lingual obstruction occurred and no amount of further resection could improve the collapsibility (100). Palatal implants with or without tongue stiffening did not change the Pcrit. While stiffening of the tongue *via* implanting in the same direction as the genioglossus muscle fibers and in the C region of the tongue (adjacent to the soft palate), without any palatal component, gave the best results in the reduction of collapsibility (−7 cmH_2_O) (100). Similar to MAD therapy, these studies again emphasize the value of baseline Pcrit in response to prediction and in the follow-up of OSA therapies.

##### Hypoglossal Nerve Stimulation

A study on 5 patients with hypoglossal nerve implants (Pcrit from −1.32 ± 1.97 to – 5.30 ± 3.30 cmH_2_O) and 9 patients with fine-wire electrodes inserted into the genioglossus muscle (Pcrit from 1.63 ± 2.02 to −1.56 ± 2.53 cmH_2_O) showed a reduction in the collapsibility and an increase in airflow when the electrodes were stimulated, unrelated to the anatomical site of the collapse. Furthermore, the reduction in AHI in patients with implants was significantly associated with a decrease in collapsibility (20). Furthermore, some preliminary studies on animals with stimulation at the distal ends of the hypoglossal nerve demonstrated a reduction in collapsibility (−3.4 ± 0.8 to −8.3 ± 1.7 cmH_2_O) and an increase in maximum airflow (211 ± 33 to 465 ± 25 ml/s). However, stimulation of the proximal ends had no effect on either the collapsibility or the airflow (101).

In a multimodal model, taking into account the four pathophysiological traits and their interactions, hypoglossal nerve stimulation (HNS) showed a lower efficacy in patients with milder collapsibility, defined as passive ventilation. This might be explained by the presence of other non-anatomical underlying traits that are responsible for OSA in this preselected cluster of subjects with OSA s (102). Nonetheless, effective HNS, significantly reduces collapsibility in the responders (20).

##### Combination Therapy of Genioglossus Stimulation and Mandibular Advancement

The combination of genioglossus muscle stimulation and MAD therapy has a synergic effect on the improvement of OSA and in the reduction of collapsibility (103). In 14 anesthetized patients with OSA, the baseline Pcrit (2.9 ± 2.2 cmH_2_O) was reduced from 0.9 ± 2.5 cmH_2_O after genioglossus muscle stimulation to −1.4 ± 2.9 cmH_2_O after mandibular advancement (to the level of maximum mandibular advancement) and to −4.2 ± 3.3 cmH_2_O in the combination of both the therapies (103).

##### Uvulopalatopharyngoplasty

Evaluation of Pcrit in subjects with OSA, before and after UPPP suggested that the level of Pcrit before the surgery is not predictive of the response to surgery (104). However, the amount of decrease and degree of change in Pcrit after the surgery compared to its baseline, is associated with a good response to surgery. Baseline disordered breathing rate from 71.1 ± 22.4/h before surgery was decreased to 44.7 ± 38.4/h afterward. This was correlated with a significant decrease in Pcrit from 0.2 ± 2.4 to −3.1 ± 5.4 cmH_2_O. A closer look at the intervention in the responders vs. nonresponders showed that the collapsibility was significantly reduced (−0.8 ± 3.0 to −7.3 ± 4.9 cmH_2_O). In contrast, the collapsibility did not change in the non-responders (1.1 ± 1.6 to 0.6 ± 2.0 cmH_2_O). The resection of tonsils caused a greater reduction in collapsibility (Pcrit in patients with −4.8 ± 5.9 vs. without −2.0 ± 1.9 cmH_2_O tonsillectomy). The researchers suggested that the amount of tissue resection and the anatomical level of collapse might play a role in the effect of the therapy on Pcrit (104).

##### Tonsillectomy and Adenoidectomy

Tonsillectomy and adenoidectomy are used often in children who suffer from OSA. These treatments significantly decreased the collapsibility after the procedure (from 1 ± 3 cmH_2_O to −7.2 ± 4.0 cmH_2_O) and reduced the airway resistance [4.3 (1.5–10.3) to 2.8 (1.7–4.7) cmH_2_O /L/s] (74, 75).

#### Pharmacologic Options

There are several pharmacological options to treat OSA. Acetazolamide reduces the breathing events and increases the inspiratory drive (105, 106). However, acetazolamide does not affect the collapsibility (107). Recently a combination of a noradrenergic drug atomoxetine plus the antimuscarinic oxybutynin has been shown to reduce the severity of OSA (108). Both the combination of atomoxetine-oxybutynin and each medication alone improved the collapsibility of the pharyngeal airway (109). Trazodone increases the respiratory arousal threshold in subjects with OSAs without affecting the pharyngeal collapsibility (110).

## Discussion

To the best knowledge of the authors, this is the first review on Pcrit and OSA in the current format. Pcrit is a measure of collapsibility in the pharyngeal airway. Prior studies have noted the importance of this measurement in achieving a better understanding of the underlying factors and the severity of OSA. It helps categorize patients with OSA, which in turn improves the treatment and response prediction. Pcrit has correlations with and is affected by various physiological factors and treatment modalities. The new paradigm toward targeted OSA therapy in accordance with precision medicine principles and personalized medicine is the aim of many of the new endotyping projects on OSA. One of the most important components of any emerging endotyping model is collapsibility and thus Pcrit. This shows the importance of having a good understanding of the best, as well as, the easiest method to have data over this feature in routine clinical practice.

The more positive the Pcrit is, the more collapsible the pharynx is. In normal adults, Pcrit is estimated to be around −13.3 ± 3.2 cmH_2_O (22). In patients with OSA, more positive values are present, approximately around 2.5 ± 1.0 cmH_2_O and almost never below −5 cmH_2_O (19, 60). In current clinical practice, the severity of OSA is defined by AHI. However, AHI is not sufficient as it gives an estimate of the frequency of pharyngeal obstructions yet does not take into account the severity of these obstructions (111). Pcrit gives an estimate of the forces that cause these obstructions (111).

Enlargement of pharyngeal soft tissue in proportion to the bone (64, 65), obesity (60, 63), and an inferiorly located hyoid which results in a longer pharyngeal airway (63), neck flexion (12), supine positions (12, 23, 55–57, 66), male gender (57), aging (78), and sleep fragmentation (88), are all correlated with higher collapsibility and more positive Pcrit values. The effect of the length of the pharynx might be explained by Poiseuille's law. An increase in the length of a tube is one of the factors that contribute to flow retardation through a collapsible tube. Thus, an inferiorly located hyoid which results in a longer pharyngeal airway results in an increased resistance to flow and a higher collapsibility. During infancy, the airway is highly collapsible (66, 70). This might be due to a more elastic airway with underdeveloped neuromechanical structures. From infancy to childhood, the airway becomes more stable with more negative Pcrit values (71, 72). As the child progresses to adolescence, the airway collapsibility increases again and the neuromotor reflexes which are responsible to keep the airway open become less affective (77). This reduction of protective reflexes continues till adulthood and with aging (78). Older subjects also have a longer pharyngeal airway and higher parapharyngeal fat deposition, which also contribute to an unstable collapsible airway (69, 79, 80). The findings of the study on the relationship between sleep fragmentation and Pcrit point to a vicious cycle of deterioration in subjects with OSA (88). On the one hand, breathing events cause sleep fragmentation. On the other hand, the resulted sleep fragmentations themselves, induce a more collapsible airway and hence worsen the severity of OSA.

The contradictory results of the studies on the relationship among different sleep stages and Pcrit might be due to the complicated underlying pathophysiology of OSA (12, 58). In patients with OSA, who highly rely on their dilator muscle activity for keeping the airway open, the reduction of genioglossus muscle activity during REM sleep might result in an increase in the collapsibility during REM sleep in comparison to other sleep stages (33).

The degree of baseline collapsibility can guide the choice of therapy and response prediction for various treatment modalities, such as MAD or hypoglossal nerve stimulation (95, 102). There is also a strong relationship between a reduction in collapsibility and various treatment options. Weight loss (91), CPAP (12, 90), mandibular advancement (93, 94, 96, 99), palatal resection and stiffening of the soft palate (99), tonsillectomy and adenoidectomy in children (74), UPPP (104), hypoglossal nerve stimulation, and activation of the protrudor muscles (genioglossus muscle) (20, 101, 103, 112–114), all contributed to the reduction of collapsibility, and more negative Pcrit and baseline collapsibility values showed associations with the outcome of the treatment. These data point to the importance of the use of Pcrit in the prediction of response to these therapies or as a follow-up modality.

The combination of these findings provides support for the conceptual premise that pharyngeal collapsibility is one of the main contributors in the pathophysiology of OSA. Therefore, knowledge of the level of collapsibility in the patient can be very helpful (7, 24, 36–38, 94). Due to the difficulty of its measurement techniques, collapsibility is not routinely measured. The earliest Pcrit measurement methods of data acquisition, where the expert performs long pressure drop for the whole duration of the night that needs breath-by-breath evaluation of the airflow and pressure measurements, are very accurate; yet, they are labor-intensive and time-consuming (12, 18–20, 25). The “abbreviated method” enables a unified simpler method for measurements (23). It shortens the duration of pressure drop and gives a defined protocol for Pcrit measurement. This method also allows the clinicians to perform various measurements in one subject just during one night. In this way, the researcher can explore the effect of the position, treatment modalities, and other affecting factors on Pcrit in one night. Despite the superiority and ease of performance compared to the earlier methods, the abbreviated method still requires extensive data processing techniques involving breath-by-breath evaluation. To reduce the time required for the analysis, the “simple method” was devised. In this method, instead of the breath-by-breath evaluation, the range of nasal pressure over which the inspiratory airflow amplitudes are markedly varied and the flow is limited, is isolated. This flow-limited segment is used for calculating the Pcrit (29). Despite being advantageous and quicker, the “simple method,” cannot be used in routine practice, as it still requires expert signal analysis.

To overcome the limitations of natural sleep, awake state surrogates and Pcrit during drug-induced sleep were investigated (18, 93, 115–118). Upper airway collapsibility index, as measured by the application of negative pressure pulses in brief periods during wakefulness, showed to have a strong association with Pcrit and might be a viable marker of pharyngeal collapsibility level (37). Some studies suggest that anesthesia causes an increase in the pharyngeal collapsibility and that it might not be possible to accurately perform Pcrit measurement during anesthesia (30, 31, 76). However, the comparison of Pcrit measurement during natural and drug-induced sleep in healthy subjects and in subjects with OSAelucidated no difference between the two (18, 93, 115–118). While anesthesia overcomes the shortcomings of natural sleep and has the added advantage of performing the measurements during daytime working hours, further investigations under anesthesia with larger sample sizes are required to confirm the results of these studies.

The non-invasive surrogate techniques, such as the prediction of Pcrit level by FEM (43), or the estimation of the pharyngeal collapsibility using the peak inspiratory flow from a normal overnight PSG (24), and other mathematical models of pressure/cross-sectional area (47, 52), are promising techniques for having data on the level of collapsibility of patients in routine clinical practice. The use of digitally reproduced models of the airway, as is done by FE modeling, has the advantage of testing the effect of various therapeutic and physiologic changes in one subject under different conditions (99). In clinical practice, it is not possible to test how both partial or complete resection of an anatomic structure affects the collapsibility of the subject under otherwise similar conditions. However, FE modeling provides the means to do so. One of the disadvantages is the needed level of expertise to reproduce a model. Another downside is the required cost and time for using the imaging techniques and in the production of models. These techniques depend on the quality of the acquired images, for example, a dynamic MRI does not always provide high-quality images. Therefore, they are difficult to apply in larger cohorts (16, 43).

Azarbarzin et al. (24) estimated the pharyngeal collapsibility using the peak inspiratory flow from a normal overnight PSG. This study demonstrated a strong correlation between active Pcrit and peak inspiratory and mid inspiratory airflow, independent of the AHI. No correlation with passive Pcrit was shown. This might be due to the effect of the sleep stage or the study setting. The main advantage of this technique is that it allows for Pcrit calculation without additional measurements; yet, further research with a large sample size is needed to make this available in routine clinical practice.

The gold standard measurement techniques have few limitations that should be considered. During gold standard Pcrit measurements, an esophageal pressure catheter is used for categorizing the flow-limited breaths into obstructive or central. However, as the esophageal catheter is inserted through the nostril and passes the collapsible segment of the airway, it might affect the Pcrit levels. To address this apprehension, a study showed that the esophageal catheter used to measure the respiratory effort had no effect on the final results of the measured Pcrit (119). The collapsibility was measured with and without the catheter and no significant difference between the Pcrit was found between the two (with: −1.5 ± 5.4, without: −2.1 ± 5.6 cmH_2_O) (119). Additional limitations of the current measurement techniques for collapsibility are the issue of variability among the applied techniques, the need for an expert to conduct these measurements, and due to its dependency on the examiner (12, 17–20, 23, 25, 29).

## Conclusion

Critical closing pressure is the gold standard measure of the degree of collapsibility of the pharyngeal airway. It is affected by many physiologic factors and treatments. The baseline Pcrit value is an essential part of categorizing patients with OSA into various endotypic groups, which subsequently improves the response prediction, upfront therapy selection, and the follow-up of therapy options.

There are various methods for the measurement of the level of collapsibility. The earlier gold standard methods of data acquisition are all invasive, time-consuming, and are mainly performed during natural sleep. Pcrit measurement during induced sleep or awake surrogate parameters might overcome the shortcomings of natural sleep and has the added advantage of performing the measurements during daytime working hours. The non-invasive surrogate techniques, such as the prediction of Pcrit level by FE modeling or the estimation of the pharyngeal collapsibility using the peak inspiratory flow from a normal overnight polysomnography, are promising techniques for having information about the level of collapsibility.

These results show that the measurement of Pcrit in routine clinical practice is important. Therefore, it should not only be measured in research environments, but clearly it also has a potential role in the routine clinical assessment of patients with OSA. However, at this point, there is no unified method to measure Pcrit in routine clinical practice, and further research is needed to arrive at a uniform, reliable measurement method in routine clinical practice.

## Author Contributions

EK, SO, and OV worked on the conception. EK performed the analysis, the review of articles, and drafted the final version of the article and figures. EK, EV, SO, and OV contributed to the first draft of the manuscript. EK, EV, MD, MW, JV, SO, and OV contributed to the revision and final approval of the manuscript. All authors contributed to the article and approved the submitted version.

## Conflict of Interest

MD holds a senior postdoctoral fellowship at the Research Foundation Flanders (FWO) (12H4520N). JV reports grants from SomnoMed, AirLiquide, Vivisol, Mediq Tefa, Medidis, OSG, Bioprojet, Desitin, Philips, and ResMed outside the submitted work. SO holds a postdoctoral fellowship at the Research Foundation Flanders (FWO) (1299822N). OV holds a Senior Clinical Fellowship Grant (Fundamenteel Klinisch Mandaat) from the Research Foundation Flanders—Vlaanderen (FWO) (1833517N) and reports grants from Philips and Somnomed at Antwerp University Hospital and from the Inspire Medical Systems, Nightbalance, GSK, and Liva Nova at the Antwerp University Hospital outside the submitted work. The remaining authors declare that the research was conducted in the absence of any commercial or financial relationships that could be construed as a potential conflict of interest.

## Publisher's Note

All claims expressed in this article are solely those of the authors and do not necessarily represent those of their affiliated organizations, or those of the publisher, the editors and the reviewers. Any product that may be evaluated in this article, or claim that may be made by its manufacturer, is not guaranteed or endorsed by the publisher.

## Abbreviations

AHI: apnea-hypopnea index
BMI: body mass index
Cm of H_2_O: centimeter of water
CPAP: continuous positive airway pressure
DISE: drug-induced sleep endoscopy
ECG: electrocardiography
EEG: electroencephalography
EMG: electromyography
FE: finite element
MAD: mandibular advancement device
OSA: obstructive sleep apnea
Pcrit: critical closing pressure
PSG: Polysomnography
RDI: respiratory disturbance index
REM: rapid eye movement sleep
UPPP: uvulopalatopharyngoplasty.
